# How hard can it be to include research evidence and evaluation in local health policy implementation? Results from a mixed methods study

**DOI:** 10.1186/1748-5908-8-17

**Published:** 2013-02-12

**Authors:** Bridie Angela Evans, Helen Snooks, Helen Howson, Myfanwy Davies

**Affiliations:** 1 CHIRAL, ILS2, College of Medicine, Swansea University, Swansea, SA2 8PP, UK; 2 Medical Directorate, Welsh Government, Cardiff, Swansea, UK; 3 School of Social Science, Bangor University, Bangor, UK

**Keywords:** Health policy implementation, Evidence-based policy, Research and evaluation, Chronic conditions management, Health service delivery

## Abstract

**Background:**

Although an evidence-based approach is the ideal model for planning and delivering healthcare, barriers exist to using research evidence to implement and evaluate service change. This paper aims to inform policy implementation and evaluation by understanding the role of research evidence at the local level through implementation of a national chronic conditions management policy.

**Methods:**

We conducted a national email survey of health service commissioners at the most devolved level of decision-making in Wales (Local Health Boards – LHBs) followed by in-depth interviews with representatives of LHBs, purposively selecting five to reflect geographic and economic characteristics. Survey data were analysed descriptively; we used thematic analysis for interview data.

**Results:**

All LHBs (n = 22) completed questionnaires. All reported they routinely assessed the research literature before implementing interventions, but free-text answers revealed wide variation in approach. Most commonly reported information sources included personal contacts, needs assessments, information or research databases. No consistent approach to evaluation was reported. Frequently reported challenges were: insufficient staff capacity (17/22); limited skills, cost, limited time, competing priorities (16/22); availability and quality of routine data (15/22). Respondents reported they would value central guidance on evaluation.

Five interviews were held with managers from the five LHBs contacted. Service delivery decisions were informed by Welsh Government initiatives and priorities, budgets, perceived good practice, personal knowledge, and local needs, but did not include formal research evidence, they reported. Decision making was a collaborative process including clinical staff, patient representatives, and partner organization managers with varying levels of research experience. Robust evaluation data were required, but they were constrained by a lack of skills, time, and resources. They viewed evaluation as a means of demonstrating that targets had been met.

**Conclusions:**

There is a gap between evidence-based aims of national health policy and how health services are commissioned, implemented, and evaluated at local level. Commissioners and managers are unable to routinely incorporate research evidence. If health services research is to identify most effective ways to implement high quality care, it should be incorporated into commissioning and service delivery. Local commissioners and managers need to build the critical use of research evidence and evaluation into health policy implementation at local level in order to provide consistent and effective healthcare services.

## Introduction

Evidence-based policy making sits beside evidence-based medicine as an ideal model for implementing healthcare services [1]. Research can help policy makers, clinicians, practitioners, and healthcare managers identify where improvements are needed, evaluate existing systems, and develop new policies and services based on cumulative learning from failures and success [2,3]. Including research evidence within policy implementation can enable decision makers undertake the ‘judicious application of best current knowledge’ [4].

However, there are challenges in achieving this ideal [5,6]. It is acknowledged that there are barriers both to the uptake of research findings in practice—the second gap in translation [7]—and to rigorous service evaluation and audit at local levels [8]. Improving population health outcomes relies on implementation of findings from clinical and health services research, yet transfer of research findings into practice is unpredictable and can be slow and haphazard [9]. This has led to concerns about equity of provision and access to services [10].

Some commentators describe a gap between the needs of policy and decision makers at national level, who must take account of population health needs and political priorities, and the motives of researchers who prioritize the scientific process of generating research [4,11]. Others say the mismatch lies between policy rhetoric at a national level and the reality of implementing change at local level [12]. It is known that decision makers struggle to locate and assess relevant information and to decide what information can be deemed evidence [13-15]. The development of innovative tools to summarize and synthesize research evidence to support implementation is one approach to overcoming the research-practice gap [16]. Policy makers are reported to find personal contact, timeliness, relevance, and the inclusion of summaries with research-based policy recommendations helpful. Absence of these alongside mutual mistrust and struggles about power and budget restrict the use of evidence [8].

Contextual factors, such as financial constraints, lack of trust in the value of local research, and political influences, affect whether research is used in policy making [17]. Services and staff can be constrained by policy and funding decisions which are based on misunderstandings of their working environment [18]. Health practitioners underuse research-based information [19] while health service managers report insufficient time and expertise to participate in research or don’t perceive a benefit from such activities [20]. Decision makers who develop and implement policies at local level need to perceive the relevance, cost-effectiveness, effectiveness, and implications of evidence-informed national guidance before it is followed [21].

Studies indicate that improved communication and shared understanding is necessary to make research more relevant and to clarify what commissioners and managers need from academics [8,22]. The greatest opportunity for effective evidence-based decision making may result from joint or parallel working between researchers and those developing, managing, and delivering policy and services, plus a broader understanding of concepts of evidence and policy, it is reported [5,20,23].

Against this background, researchers in two Welsh universities were asked by Welsh government policy makers to develop an evaluation strategy for a new evidence-based policy to manage chronic conditions alongside policy implementation. Chronic disease represents a significant and increasing impact on health and social care services in the developed world [24]. The highest United Kingdom (UK) rates are in Wales, where at least one-third of adults and two-thirds of those aged over 65 report having at least one chronic condition [25]. A new policy to improve management of chronic conditions services was launched by the Welsh government in 2007 targeted at those with, or at risk of developing, chronic conditions. The objectives were to delay onset or deterioration; improve quality of life and ability to self-manage; and to reduce the burden on the National Health Service (NHS) and social care services [26,27]. The policy called for a ‘clear, consistent evidence-based approach to chronic conditions management’ [26], and set out a model of care based on UK and international evidence. The requirement to undertake monitoring and evaluation was an explicit stage in the implementation cycle, to inform needs-based service planning and quality assessment. It also demanded ‘strong leadership, courage and determination at all levels’ to achieve the clear targets listed in a subsequent Service Improvement Plan [27]. These ranged from completing needs assessments, strategies, and partnership plans, appointing core chronic conditions management (CCM) community teams, developing community care services and self care programmes, and having effective data management systems for monitoring and evaluation. Responsibility for implementation lay with local health boards (LHBs). These organizations were in charge of commissioning at the most devolved level of local decision making. They had responsibility for local primary and secondary health services and were coterminous with local authorities to aid collaboration. In order to deliver the chronic conditions policy, LHBs were required to work in close partnership with local authorities, the voluntary sector, and patients. Staff had responsibility for translating the policy and targets into action within the local contexts of their respective health board areas and for planning evaluation.

Although there are reported challenges to uptake of research findings in policy making—the macro level—it is not known how far this extends towards implementation and service delivery—the meso and micro levels [28]. Examining whether research evidence is used and how it is incorporated into decision making provides valuable information to understand the process of health policy implementation. Against a health policy background that highlighted research and evaluation, we describe the status and use of research evidence in applying and delivering a prominent health service strategy. We use these findings to discuss how research evidence and evaluation may be routinely used in local implementation of other health policies.

### Aim and objectives

The aim of this study was to understand the role of research evidence at the local level where national health policy is interpreted and implemented in the form of service delivery models and interventions received by patients.

Objectives were: to identify influences on decision-making by commissioners and service managers when implementing policy; to describe approaches to undertaking evaluation of policy implementation at local level, and challenges experienced; and to identify support needed to undertake evidence-based implementation and evaluation.

## Methods

In this study, we used a mixed-methods approach incorporating sequential data collection. We administered questionnaires to all 22 LHBs in Wales, then carried out semi-structured interviews with a sample of LHBs to enhance interpretation of survey data [29]. We administered questionnaires in order to gather an overview of attitudes and experiences within all local health boards. The purpose of the interviews was to explore in further depth the issues raised by survey respondents.

We collected survey data from all 22 LHBs. These organizations commissioned primary and secondary healthcare services and were responsible for implementing the chronic conditions services policy. This survey was conducted before reorganization in 2009 restructured LHBs and reduced the number to seven.

The survey and interview guide were structured in line with our study objectives. We developed a structured questionnaire with some spaces for open-ended responses in order to gather data on perspectives and approaches towards implementing the new chronic conditions policy, including use of research evidence. Questions related to our study objectives and covered: decision making when commissioning and implementing policy; and approaches to research and evaluation including barriers and facilitators.

We circulated the questionnaire to all LHB Chief Executives by email, with a request to pass it on for completion by the person responsible for implementing and managing chronic conditions services. We included information explaining that survey comments would inform development of a framework to evaluate implementation of the new Chronic Conditions Management (CCM) policy at national and local levels. We sent reminders to non-respondents by email and made telephone calls to maximize response rates.

In-depth interviews were then conducted by two researchers (BAE, MD) with representatives of a sample of LHBs to explore in more depth the experiences and attitudes reported by commissioners and managers in the questionnaires. We interviewed representatives from one in four LHBs in order to gain a range of views. The LHBs were selected purposively to reflect different geographic (rural/urban) and economic (deprived/affluent) characteristics and different sized health boards. We approached survey respondents or other senior staff with responsibility for overseeing or implementing the chronic conditions services to participate in the interviews. We invited respondents by email and made follow-up telephone calls to confirm arrangements. All those contacted consented to interview (n = 5) so that the sample adequately represented the sampling strata [30,31]. Interviews were undertaken face-to-face, or by telephone where contact in person could not be arranged. Interviews were tape recorded and detailed notes also made by the interviewer. We designed the semi-structured interview schedule in line with our study objectives, to expand on the questionnaire data by further examining influences on decision-making, identifying decision-makers, and exploring how their own activities contributed to the evidence base. Before conducting the interviews, we viewed questionnaire responses in order to be able to develop and explore respondents’ comments and any contradictions.

We followed principles and standards of ethical research although formal approval was not required for this study because it was classified as a service evaluation. We obtained informed consent from all interview participants. All study data were anonymized and stored securely.

Data from the closed survey questions were managed using Excel. We analysed survey data descriptively while open question responses were analysed using a framework developed from the study objectives and interview topic guide [32,33]. Two researchers (BAE, MD) studied the interviews, and identified and coded relevant parts of the interviewees’ responses within the framework’s headings. They discussed modifying themes and categories after considering all transcripts but chose to maintain the original headings and groupings because they agreed that the results consistently related to them. They then discussed key issues and areas of similarity and difference in order to agree results, which were structured against study objectives.

Because interview responses expand on the questionnaire replies, we present the questionnaire and interview results together. Results are presented against each study objective because these underpinned the structure of all data collection and analysis. We present quantitative survey results using simple frequencies and proportions and give examples of free text responses. Quotations have been selected and reported to illustrate the interview results. They reflect the majority views, unless identified as describing an uncommon response. Questionnaire free text responses are identified with the LHB identification number preceded by the letter Q (*e.g.*, Q1); interview responses are identified by the letters IR and the identification number (*e.g.*, IR1).

## Results

### Response rates

We received completed questionnaires from all 22 local health boards: 13/22 were completed by Directors or Chief Executives; 9/22 were completed by senior nurses and CCM managers/coordinators. We undertook five interviews with six staff members of five LHBs including five senior CCM service managers and a Chief Executive. Respondents held responsibility for putting policy into operation and commissioning services and their remit included resource allocation, service development, and implementation.

### Influences on decision-making

When implementing new policies, respondents described a decision-making process that incorporated information from a variety of sources. All questionnaire respondents (n = 22) said they routinely assessed the evidence base before implementing new policies, although the term evidence was widely interpreted. One-half of respondents reported basing decisions on multiple information sources, including National Public Health Service information or research databases alongside locally collected data or personal contacts. Questionnaire respondents did not report using high-grade research evidence sources, such as systematic reviews or meta-analyses, although respondents Q16 and Q5 listed literature reviews among the information sources accessed in their organizations. Nor were there any references to national guidance in the questionnaire replies. Free text responses (see Table 1) defined evidence as information derived from contacting other commissioners/project managers; involving service users within multidisciplinary working groups; and undertaking local needs assessment, evaluation, or service reviews.

**Table 1:** Free-text questionnaire responses describing information sources used in commissioning

**Question: When new services or service changes are planned, do you routinely assess the evidence base?**	
Q6	We would undertake a full search of recent evidence online and also attempt to speak to other areas in the UK who have undertaken similar projects to benchmark and share good practice.
Q12	Other LHBs have approached as to their experiences with CD [compact disc], articles and evidence base been gathered (*sic*). Groups and multi-agency/multidisciplinary colleagues have been consulted.
Q13	For interventions we would look to standard databases *e.g.,* Medline. For service models we use Web Fetch to search examples from other NHS organizations.
Q16	Data from NPHS, literature reviews available/best practice. Hear from internal information, paper published, attending a conference.
Q18	New services considered by disease specific group with clinical or managerial lead, then passed to Steering Group attended by medical director and director of public health.

Interview respondents described in more detail the range of influences that informed decisions about commissioning and implementing services under the new chronic conditions policy. They did not report that research evidence was included in those decision-making processes. Instead, all the respondents explained how government policy and initiatives were among the greatest influences. Respondents reported that targets framed the commissioning context and drove decisions at all levels. In their efforts to meet these requirements, they said that their organizations were quick to adopt any initiative, wholesale or piecemeal, if it appeared to offer performance benefits. Interview respondent one dubbed this as ‘jumping on any bandwagon’ (IR1).

They also reported that budgetary issues were influential in their commissioning choices, especially in a tough financial climate. For example, money could be linked to grant projects, which made commissioners feel forced to make decisions for financial reasons, even if for the short term. They felt that finance was often also linked to government priorities and targets. Two interview respondents said decisions on new services were made on the basis of a business case.

Interview respondents reported that evidence of need, as illustrated by needs assessment reports or routinely collected local data, was used to inform strategic and specific decisions. At one LHB, the respondent said staff had confidence in these data and the review process. More generally however, other respondents acknowledged that this information was of weak quality and questionable relevance. Shared knowledge and expertise among professional colleagues was also reported to play an important part in decision-making. This reliance on networking was referred to as ‘responsive practice’ by one respondent (IR4). A senior manager said she had implemented a particular care approach in her LHB because of her personal preference that was informed by professional contacts and not according to the national policy and perceived local needs. Another respondent listed a number of pragmatic reasons, including external and internal organizational factors, that had informed their planning and decision making. While the imperative for action on implementation and service delivery was said to be strong, the respondent summarized the process as having ‘no coherent plan on the ground… the approach is fragmented’ (IR2).

Interview respondents said that decisions about using research evidence were also influenced by the role and experience of the decision takers. All respondents said that people with little or no research experience and varying levels of clinical experience played a key role in decision making across commissioning structures. These decision makers were said to include clinical staff, patient representatives, and senior managers from different organizations working together in forums. These forums included: a steering group of general practitioners (GPs), nurses, consultants and patient representatives advised by specially-appointed task and finish groups; a management group considered by the respondent to be without clinical or evaluation experience; and a partnership of LHB, NHS Trust, and local authority representatives.

### Approaches to undertaking local policy evaluation and challenges experienced

Table 2 presents questionnaire responses about LHBs’ experience of, and plans for, evaluation. In response to the question inviting them to describe their LHB’s overall approach to research and evaluation, 21 respondents stated that they would include data relating to structure, processes, and outcomes in a typical evaluation, with the other LHB answering they would not include any of these. In free text replies, respondents reported that approaches to undertaking research varied, from ‘ad hoc’ (Q10) to ‘integral’ (Q14) but that it was rarely done in collaboration with other LHBs. One recorded that two attempts to initiate a local research network had failed; six respondents admitted that little or no research was used or available locally. Most respondents (15/22) reported that they were encouraged to build research and evaluation into service delivery or development. Free text responses revealed how the approaches to evaluation varied. Respondents reported that they had commissioned an evaluation of a service redesign, collaborated with a university, or undertaken internal reviews or audit. Other examples of their approaches to undertaking evaluation were given as follows: developing a research and development strategy; adhering to research governance process; working with GP and nurse research fellows; working with a service evaluation group; and collaborating with a voluntary group.

**Table 2:** Responses to questions about LHBs’ experience of, and plans for, evaluation

**Question**	**Yes**	**No**	**Don’t know**
Were you aware of the forthcoming model for Chronic Conditions Management (CCM) produced by the Welsh Assembly Government, prior to receipt of this questionnaire?	21	1	
In evaluating or monitoring a typical project, would the following types of data be used?			
o Data relating to structure (*e.g.*, facilities, resources, skills etc.)	21	1	
o Data relating to processes (*e.g.,* care delivered, time intervals, locations of delivery etc.)	21	1	
o Data relating to outcomes (*e.g.,* health indicators, satisfaction, mobility etc.)	21	1	
Do you have plans to evaluate existing and new initiatives in CCM?	21		1
When new services or service changes are planned, do you routinely assess the evidence base?	22		
Would you be prepared to work with other LHBs to evaluate initiatives on a wider scale?			
o Nationally	20	2	
o Regionally	21	1	

In the questionnaires, all LHBs reported that they intended to evaluate the CCM programme locally. One-third of responses (n = 7) suggested these plans were in hand by listing services to be evaluated and methods proposed. These included a longitudinal patient study, monitoring against performance indicators, before-and-after survey, and analysis of routine data.

Questionnaire respondents all reported challenges to undertaking local evaluations (Table 3). These included lack of confidence concerning evaluation planning and concerns about availability and quality of data. They acknowledged a lack of statistical and evaluation skills, which they said impacted on their ability to plan and undertake research. For example, Q4 reported ‘difficulty in identifying outcomes that are robust and universally accepted’ and Q1 stated it was ‘difficult to determine which data are suitable for measuring impact.’ Respondents also reported that they lacked the time and finances to undertake evaluations. These limitations were reported to impact on the number of planned evaluations and quality of research because, as Q15 reported, it was ‘done within current resources so often not as robust.’

**Table 3:** Challenges faced locally by LHBs when carrying out evaluation

	**Challenge**	**Not challenge**
Insufficient staff capacity	17	2
Limited skills	16	4
Cost of evaluation	16	3
Limited time	16	1
Competing priorities	16	3
Availability of routine data	15	
Lack of clarity about purpose	4	9
Other	4	

Interview respondents provided more detail about approaches to evaluation. When undertaking evaluations, interview respondents said that they used a mix of quantitative data, generally focusing on admissions rates and clinical measures, as well as measures of patient experiences and quality of life. Interview respondent four said evaluation was generally undertaken quickly and retrospectively, relying on routine and survey data, often using validated tools. Some external evaluations had been commissioned by LHB5 to supplement in-house research measuring change in relation to baseline data. However, respondents were uncertain how to proceed with evaluation when these data were not available. Interview respondents said they supported a rigorous approach to undertaking evaluation within their organizations and were glad to carry out, or commission, some studies, but acknowledged there were instances where limited or no evaluations were undertaken. In some cases this was because data, skills, or resources were not available. One respondent said they generally undertook evaluation if it was feasible, although other respondents reported that there were no routine systems for evaluating the services for which they carried responsibility. Variable access to data meant that, even when undertaken, evaluation did not necessarily report what they felt was useful information, respondents said:

‘a major step limiting effective research and evaluation…process can’t be the proxy for outcomes…if you are going to do robust research and evaluation, you are going to stop at the first door.’ (IR5)

There also appeared to be a tension between the demands of delivering and accounting for services and the independence and rigour of a research approach. Respondents reported that commissioners told them they wanted robust information to enhance decision making in a tight financial climate, but in practice this demand for high-quality evaluation could not always be met because of the lack of evaluation skills among staff and competition for limited time and financial resources. They also said there were different understandings within their organizations of the purpose of evaluation, both strategically and at practitioner level. Respondent three saw it as a rigorous approach where ‘independence gives objectivity…not to be pulled by your heart strings’ (IR3). Respondent one said there was a conflict of priorities for nursing staff tasked with providing care and also collecting evaluation data. Meanwhile, s/he was planning a service evaluation, but was not confident that the organization would be interested in patient experiences and health outcomes rather than measurements against centrally defined targets. Interview respondents felt that all local health boards experienced the tensions between service delivery and rigorous research. As a result, they said they had low confidence in evaluation findings reported by other LHBs because they perceived them to be driven by the imperative to prove the targets had been met, as respondent four noted: ‘The value of some of the evaluations and the quality is not good.’ (IR4)

### Support needed to undertake evidence-based implementation and evaluation

Questionnaire and interview respondents suggested that central guidance, technical and academic support, standardized approaches and frameworks to research and evaluation, plus additional resources would help them to carry out rigorous evaluation within their LHBs. Interview respondents said they were cautious about receiving guidance from someone who might lack knowledge and understanding of local health boards. They identified opportunities for better working between LHBs and with local authorities to allow joint services and evaluations and thus enable better evaluation planning and data sharing.

Areas requiring support included the development of research and evaluation questions and methods, identifying and accessing data, and undertaking analysis. Several respondents believed that data access issues were systematic; some data about chronic conditions patients were not routinely collected or linked between primary and secondary health services, or there was a long time lag until data were available. Another respondent did not have confidence in the quality of available data. They identified a need for improved skills and training but also acknowledged they had little time to receive training or undertake evaluations.

## Discussion

### Summary of findings

In this study of health service commissioners and managers, research evidence was reported to be just one influence among a range of factors that were considered in commissioning and implementing local policy. Government targets, financial imperatives, and other information usually played more important parts in these decisions. Local influences on decision making arose from the interrelationship between financial pressures, local political issues, and the need to deliver patient services at the same time as meeting local needs, delivering national targets, and putting policy into practice. A lack of skills, time, and resources limited the capacity of local decision makers to undertake evaluation of new and existing services. These factors reduced the opportunity to contribute information to help further policy and services planning. Where services were evaluated, the emphasis was on demonstrating that targets had been met in order to justify resource use. Respondents said they would value central evaluation support.

### Strengths and limitations

We received a 100% response to the questionnaires, providing a comprehensive report of Welsh LHBs’ views. The interview sample, while small, represented almost one-quarter (5/22) of organizations. We aimed to minimize risk of selection bias by our purposive sampling strategy and request to interview staff with similar areas of responsibility and holding senior commissioning and managerial roles. However, we cannot be confident we identified the most appropriate respondent or that the views of one staff member, albeit a higher-level decision maker in their organization, could represent the whole organization. Additionally, the small sample size does limit generalizability of results to other populations and settings, and results should be interpreted accordingly. This study was strengthened by our mixed-method approach incorporating questionnaires and interviews. Questionnaire results gave an overview of attitudes and experiences within local health boards, although only one survey was completed per organization. The interviews illuminated survey responses by allowing us to explore issues in further depth. This enhanced our interpretation of study findings.

Questionnaire and interview respondents in this study were selected for their role in applying policy at a local level through developing and implementing chronic conditions services. As the research team was closely involved in the process on a national level, some response bias may be expected. The reported influence of national policy agendas may have been heightened by the timing of this study, which coincided with the well publicized launch of the chronic conditions policy. Additionally, we included information with the survey explaining that results would inform the development of a framework for evaluating the policy. Nonetheless, the frankness of views expressed suggests that the relationship of the team to the nationally-driven strategy was perceived to offer participants a valuable opportunity to engage national policy makers with the conflicting demands of local policy delivery and evaluation.

### Implications

Results of this study indicate that the environment of local health commissioning and policy implementation does not support an evidence-based approach. Even when a government policy is underpinned by research findings and builds ongoing evaluation into the implementation cycle, health service managers who commission and implement policy at micro level are limited in their ability to use research evidence and perform appropriate evaluation. In addition to the known lack of evidence-based policy making at a national level and in other policy making arenas [34-36], our study has identified a gap between the evidence-based aims of national health policy and the practice of commissioning, implementing, and evaluating health services locally.

Black suggests the relationship between research evidence and policy making is weakened by competing pressures on decision makers whose goals, such as social relations or electoral considerations, are often at variance with research evidence [5]. He challenges what he calls the ‘implicit assumption of a linear relationship between research evidence and policy.’ He notes the process is more interactive but also unbalanced, with research evidence having most influence in central policy and less at local level where policy making is marked by negotiation and uncertainty.

In this study, which explored health policy implementation at local level, politically sensitive clinical and service delivery targets were set at a national level. At the same time, national-level policy also required local decisions to be evidence-based and evaluations to be objective. We observed these tensions in two phases and of two kinds. Prior to the implementation of new services, respondents described tensions between the resources required to appraise research in order to inform commissioning decisions and the resources needed to develop and introduce health services effectively and in a timely manner. Once services were in place, managers described tensions between the requirement to plan objective and effective evaluations and also to demonstrate that targets had been reached. Thus, apparently contradictory attitudes were reported which valued evidence-based policy but struggled to effectively provide and use it in practice. This gap between publically prioritizing health services research, accessing and considering evidence to make decisions was reported by Macintyre *et al.* who observed it at national level [37]. The complex issues involved in linking research evidence to decision making, plus the barriers to and facilitators of, research utilization, have also been reported in other policy fields [38-42].

Even when people with research experience were included in the local health services decision-making and implementation processes we studied, they faced multiple demands and competing priorities. Respondents could not always access research evidence and lacked the skills to assess its significance and communicate this into the decision-making forum, as has been reported at policy level [8]. Meanwhile, they distrusted the quality of research evidence generated by colleagues and contemporaries. The study also highlights the financial and political considerations that dominate each cycle of health service policy and implementation [4]. Gold says there is a ‘black box’ between the production of research and its use in policy making, and notes how much uncertainty exists in the process [43]. Our findings identify that the challenges to uptake of research findings at macro policy level are also found at this most local point—the micro level of health service delivery and implementation [28].

Within the decision-making processes described in this study, led by people with senior managerial and clinical experience, research evidence was just one among many influences. Some commentators argue that research evidence should not be the only consideration in policy and decision making, and that wider needs and values should be included [4,22,44]. Glasby and Beresford point out that the knowledge and expertise of non-academics also has a value within the commissioning and delivery process:

‘what is currently constituted as ‘evidence’ is too often dominated by academic researchers…and neglects the views and experiences of people who use and work in health and social services’ [13].

The decision whether or not to incorporate research evidence and evaluation into implementation may not be a simple either/or process. A systematic review identified that the credibility and relevance of research and the integration of findings with other information of real usefulness to health-policy makers encourages their use [11]. Respondents in this study prioritized locally relevant information sources and said that professional expertise, networking, and needs-based data were equally valid influences in decisions about implementing health services. Thus, information appears to be selected on the bases of credibility, relevance, availability, and how it relates to other priorities.

Our findings reveal that the processes of implementing and evaluating health policy at local level are complex and driven by multiple influences. Health service commissioners and managers are not able to incorporate research evidence in any standard way. Our study demonstrates that including research evidence and evaluation in policy implementation at local level is challenging and perceived to be of uncertain benefit. This raises questions about the consequence of omitting the evidence base from decisions [45]. If implementation is based on diverse, and specifically local, information, it risks being inconsistent. This could lead to variation in delivery and might ultimately affect patient care and outcomes if the most effective services are not delivered. Inefficient use of resources reduces the opportunity to provide other services to meet need [46-48].

If health services research is to identify the most effective ways to organize, manage, fund, and deliver high-quality care [49], then it should be incorporated into commissioning and service delivery processes. Local commissioners and managers need to be able to build research evidence and evaluation into local implementation processes in order to provide consistent and effective healthcare services. The use of research and evaluation in local commissioning cannot flourish when local decision makers do not access all available information sources. The heavy reliance on non-research, or ‘unofficial’ evidence, raises questions about the nature and scope of decision-making processes. Further research is needed to define this information and review its relevance.

## Conclusion

There is a gap between the evidence-based aims of national health policy and how health services are commissioned, implemented, and evaluated at local level. Commissioners and managers of local health services are unable to routinely incorporate research evidence into decision making. This study has identified that known challenges to evidence-based planning and decision making at macro level are also evident at local, or micro, levels of health services implementation. Locally sourced information is seen as more relevant evidence to inform decision making when implementing health policy and health services.

If health services research is to identify the most effective ways to implement high-quality care, it should be incorporated throughout levels of commissioning and service delivery. The consequences for equity and effectiveness of service delivery of using local evidence rather than research-based evidence are unclear. Local health commissioners and managers need to build the critical use of research evidence and evaluation into local implementation in order to provide consistent and effective healthcare services.

## Abbreviations

CCM: Chronic Conditions Management; GP: General Practitioner doctor Patients in the UK access healthcare through the GP practice with whom they are registered; LHB: Local Health Board the most devolved level of decision-making in Wales Restructuring has reduced the number from 22 to seven; NHS: National Health Service The publicly funded healthcare system in England, Scotland, and Wales.

## Competing interests

Helen Howson was Senior Health Strategy Advisor and Head of Community Health Strategy and Development at the Welsh Government when the Chronic Conditions Management policy was developed, published and implemented. Her department commissioned Swansea University to develop a Framework to evaluate implementation of the policy. The other authors declare they that have no competing interests.

## Authors’ contributions

BAE undertook data collection, analysis and drafted the manuscript. HS was study lead, developed the study, contributed to analysis and helped draft the manuscript. HH contributed to study development and helped draft the manuscript. MD undertook data collection, analysis and helped draft the manuscript. All authors read and approved the final manuscript. This study was funded by the Welsh Government.
